# Anomaly detection in chest ^18^F-FDG PET/CT by Bayesian deep learning

**DOI:** 10.1007/s11604-022-01249-2

**Published:** 2022-01-30

**Authors:** Takahiro Nakao, Shouhei Hanaoka, Yukihiro Nomura, Naoto Hayashi, Osamu Abe

**Affiliations:** 1 Department of Computational Diagnostic Radiology and Preventive Medicine, The University of Tokyo Hospital, 7-3-1 Hongo, Bunkyo-ku, Tokyo, 113-8655 Japan; 2 Department of Radiology, The University of Tokyo Hospital, 7-3-1 Hongo, Bunkyo-ku, Tokyo, Japan; 3 Center for Frontier Medical Engineering, Chiba University, 1-33 Yayoicho, Inage-ku, Chiba, Japan; 4 Division of Radiology and Biomedical Engineering, Graduate School of Medicine, The University of Tokyo, 7-3-1 Hongo, Bunkyo-ku, Tokyo, Japan

**Keywords:** Positron emission tomography, Positron emission tomography–computed tomography, Computer-aided diagnosis, Deep learning, Artificial intelligence

## Abstract

**Purpose:**

To develop an anomaly detection system in PET/CT with the tracer ^18^F-fluorodeoxyglucose (FDG) that requires only normal PET/CT images for training and can detect abnormal FDG uptake at any location in the chest region.

**Materials and methods:**

We trained our model based on a Bayesian deep learning framework using 1878 PET/CT scans with no abnormal findings. Our model learns the distribution of standard uptake values in these normal training images and detects out-of-normal uptake regions. We evaluated this model using 34 scans showing focal abnormal FDG uptake in the chest region. This evaluation dataset includes 28 pulmonary and 17 extrapulmonary abnormal FDG uptake foci. We performed per-voxel and per-slice receiver operating characteristic (ROC) analyses and per-lesion free-response receiver operating characteristic analysis.

**Results:**

Our model showed an area under the ROC curve of 0.992 on discriminating abnormal voxels and 0.852 on abnormal slices. Our model detected 41 of 45 (91.1%) of the abnormal FDG uptake foci with 12.8 false positives per scan (FPs/scan), which include 26 of 28 pulmonary and 15 of 17 extrapulmonary abnormalities. The sensitivity at 3.0 FPs/scan was 82.2% (37/45).

**Conclusion:**

Our model trained only with normal PET/CT images successfully detected both pulmonary and extrapulmonary abnormal FDG uptake in the chest region.

## Introduction

A combination of positron emission tomography (PET) using the tracer ^18^F-fluorodeoxyglucose (FDG) is a useful imaging technique to find malignant and inflammatory lesions. Computer-aided diagnosis (CAD) in ^18^F-FDG PET (hereinafter, PET) and its combination with computed tomography (hereinafter, PET/CT) has been actively studied to this day [1–8]. These CAD studies can be divided into two groups by techniques employed: (1) supervised learning and (2) semi-supervised/unsupervised anomaly detection (hereafter, anomaly detection). In the first and mainstream group [1–5], supervised learning is utilized, that is, machine learning based on a large number of images with annotations of the lesions of interest. However, preparing such annotated datasets can take a considerable amount of time [9, 10]. In the second group [6–8], on the other hand, anomaly detection is employed, that is, training only with normal class instances and detecting outliers different from the normal data [11–13]. Here, the training dataset does not require any abnormal images or lesion annotations and, therefore, far easier to prepare than annotated datasets in supervised CAD. Furthermore, such an anomaly detection CAD method has the additional advantage that it can detect any type of anomalous finding since it detects anything different from normal images. This contrasts with supervised CAD, in which detectable lesions are limited to those of the class included in the training dataset.

Previous anomaly detection method for PET or PET/CT images [6–8] has the limitations detecting anomalies only in a specific organ or region [7, 8] or requiring complicated anatomical standardization [6]. In this paper, we propose a novel anomaly detection CAD method for PET/CT images that can detect anomalies at any location in a simple way. Our method is based on Bayesian deep learning, an intersection between deep learning and Bayesian probability approaches, which can model the uncertainty of tasks as probability distributions [14, 15]. Our CAD models the probability distribution of standard uptake values (SUVs) in a normal training dataset. This allows anomaly detection by calculating Z scores, that is, the difference of the SUV from the mean in units of the standard deviation. Owing to the advantage that images can be processed in raw form in deep learning [16], our method can directly calculate Z scores at once for every pixel from a pair of PET and CT slices.

With the above as the background, in this study, we aim to develop an anomaly detection CAD system for PET/CT using a Bayesian deep learning framework. We demonstrate its feasibility by showing that it can detect both pulmonary and extrapulmonary lesions in the chest area.

## Materials and methods

### Anomaly detection

**Fig. 1:**
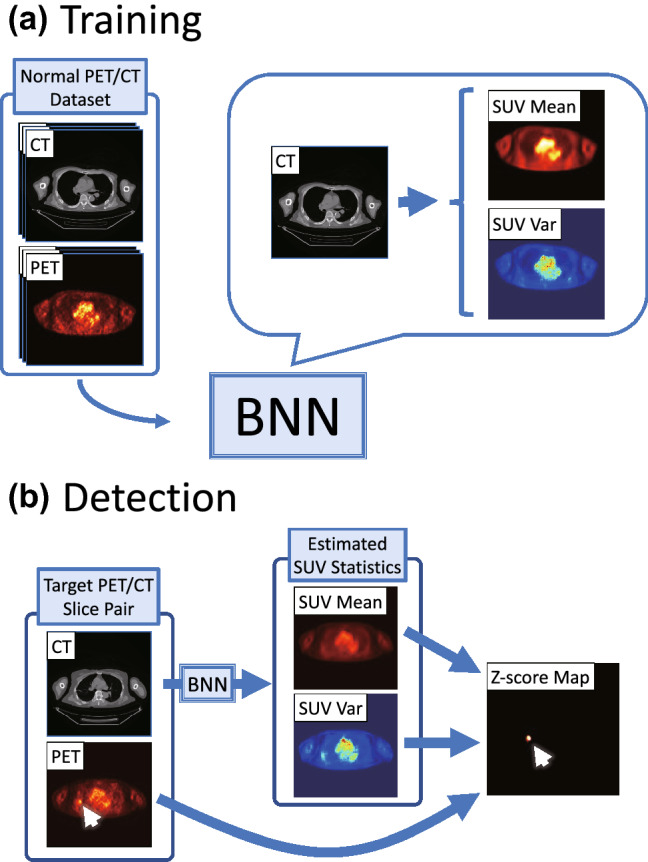
Overview of anomaly detection. **a** Training. The BNN is trained to learn the distribution of SUVs in normal PET/CT. **b** Anomaly Detection. The BNN estimates the mean and variance of the SUVs from the CT slice. The Z-score map can be calculated from these estimated statistics and the actual SUVs in the PET slice

Our anomaly detection method is performed in a two-dimensional (2D) manner: it outputs a 2D anomaly score map for an axial PET slice. The anomaly score map for the entire PET volume is obtained by simply calculating this 2D map for all PET slices independently. The overview of our anomaly detection method is shown in Fig. 1.

First, we train a deep neural network that takes an axial CT slice as the input and predicts the corresponding PET slice. This training is based on a Bayesian deep learning technique proposed by Kendall and Gal [14], so that this neural network can infer both the mean and variance of PET SUVs. That is, it provides the predictive uncertainty in addition to the prediction itself. We employ the U-Net architecture [17], which is commonly used for making pixel-level predictions. Please refer to the appendix for more details on the training and inference. Hereafter, this neural network will be referred to as the Bayesian neural network (BNN).

This BNN is trained using a dataset consisting only of normal PET/CT images. Therefore, its outputs represent the statistics of the SUV in the normal PET/CT dataset. Using these statistics, we can detect anomalies in for a PET/CT slice pair. The pixel-wise Z score of the target PET slice can be calculated from the estimated mean and variance of the PET slice as follows:

1 $${Z}_{i}=\frac{ {y}_{i}-E\left({y}_{i}\right)}{\sqrt{Var\left({y}_{i}\right)}},$$

where $i$ denotes a pixel, ${Z}_{i}$ is the Z score for the $i$-th pixel of the actual PET slice, ${y}_{i}$ is the $i$-th pixel of the actual PET slice, $E({y}_{i})$ is the $i$-th pixel of the estimated PET mean, and $Var\left({y}_{i}\right)$ is the $i$-th pixel of the estimated PET variance. The Z score represents the difference of the SUV from the mean in units of the standard deviation, and a high value indicates an abnormal FDG uptake.

### Dataset

This study was approved by the ethical review board of our institution. The subjects in this study comprised adults who visited our hospital for a whole-body medical screening program from January to October 2015. All subjects provided written informed consent that their medical images can be used for research purposes. As part of the screening program, PET/CT scans were performed on a single scanner (Discovery ST Elite, GE Healthcare, Waukesha, WI). CT images were acquired using the following parameters: field of view (FOV), 500 mm; matrix size, 512 × 512; voxel size, 0.98 × 0.98 × 1.25 mm. PET images were acquired with the following parameters: FOV, 700 mm; matrix size, 128 × 128; voxel size, 5.47 × 5.47 × 3.25 mm. These CT and PET images were resampled to an isotropic voxel size of 3 × 3 × 3 mm when used in this study. In the screening program, all these PET/CT images were interpreted in a double-reading manner: two radiologists interpret the same PET/CT image independently and the final diagnosis was determined by a discussion between them.

**Fig. 2:**
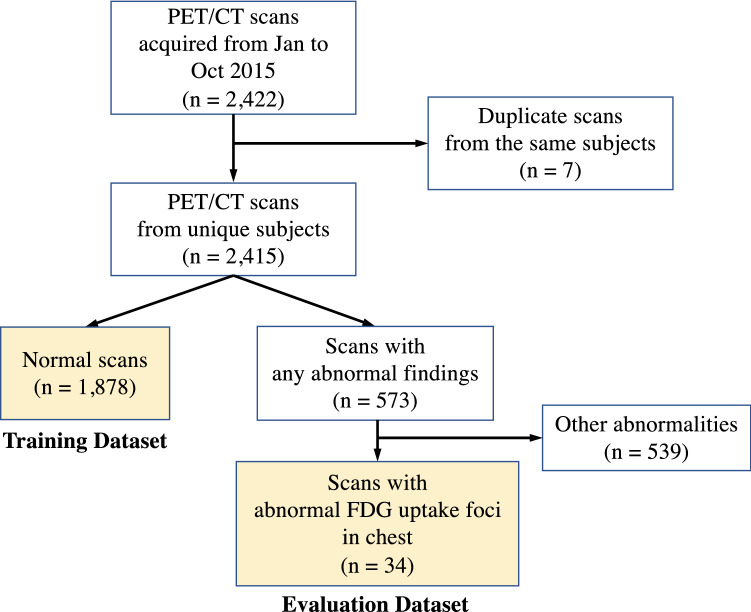
Flowchart of study inclusion

Figure 2 shows a flowchart of study inclusion. During the period above, a total of 2415 PET/CT scans were acquired and 1878 of these were determined to have no abnormal findings. Seven duplicates of scans from the same subjects were excluded so that all scans were from unique subjects. That is, if the same subject had multiple PET/CT scans during the period, only the first one within the period was used. We used all 1878 normal scans for the training of our model (1374 from males and 504 from females; mean age, 58.1 years; age range, 40–90 years). We also used the scans with one or more abnormal FDG foci in the chest region for the evaluation of our method. This evaluation dataset consists of 34 scans from unique subjects (21 from males and 13 from females; mean age, 64.4 years; age range, 41–89 years) and includes both 28 pulmonary and 17 extrapulmonary abnormal FDG uptake foci. Further details of the lesions in this dataset are shown in Table 1. A board-certified radiologist (N.H., 15 years of experience in PET/CT interpretation) annotated the locations of all the uptake foci voxel-wise.

**Table 1:** Details of the abnormal FDG uptake foci in the evaluation dataset

	Type	Number of lesions
Pulmonary	Lung Mass	9
	Pneumonia	19
	Total	28
Extrapulmonary	Lymph Node	10 (4 hilar, 3 axillary, 2 mediastinal, and 1 supraclavicular)
	Mediastinal Mass	2
	Breast Mass	2
	Bone Fracture	3 (2 clavicles and 1 rib)
	Total	17

### Performance evaluation

We evaluated the quantitative performance of our anomaly detection method at three levels: per voxel, per slice, and per lesion.

#### Per-voxel evaluation

We performed a receiver operating characteristic (ROC) analysis to evaluate the capability of voxel-wise Z scores to discriminate between normal and abnormal voxels. For comparison, we also applied ROC analysis to raw SUVs.

#### Per-slice evaluation

Similarly, the capability of our method to discriminate between normal and abnormal slices was evaluated by ROC analysis. An abnormal slice is defined as an axial slice with one or more abnormal voxels. Slice-level SUV and Z score are represented by the maximum SUV and Z score (SUV_max_ and Z-score_max_) in the slice, respectively.

#### Per-lesion evaluation

Finally, we performed a free-response receiver operating characteristic (FROC) analysis to evaluate the performance of our method in lesion localization. This FROC analysis was performed by extracting regions with a Z score greater than 3.0 as lesion candidates. Each candidate is considered true positive if and only if its centroid and that of a true lesion are within 5 mm. We also compared the performance of our method with those of the following baseline methods to show the effectiveness of Bayesian deep learning. (1) Simple thresholding: Regions with SUV of greater than 1.0 or 2.0 were considered abnormal. (2) Non-Bayesian deep learning: Using the same training dataset as above, we trained a U-net that predicts only a PET slice, without predicting variance, from the corresponding CT image. Regions that have the SUV difference of greater than 0.5 or 1.0 between the predicted and the actual PET image were considered abnormal.

## Results

**Fig. 3:**
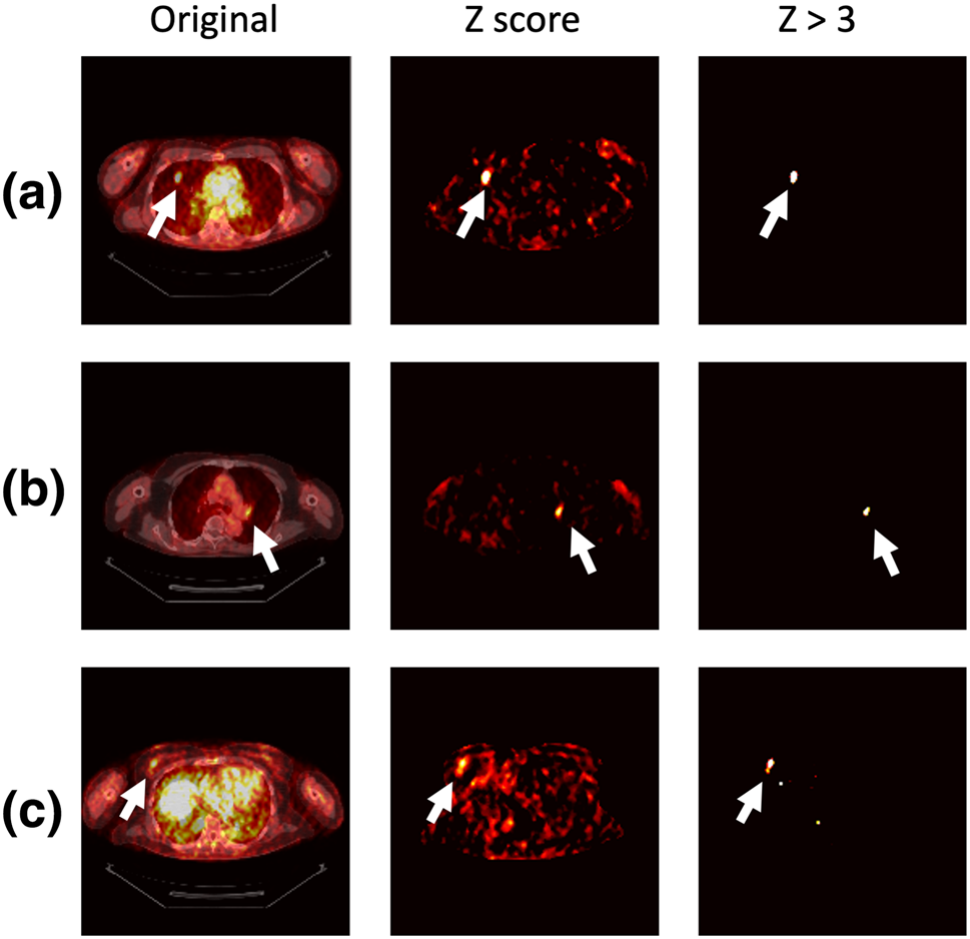
Examples of images for our anomaly detection. The original images (fused PET/CT) and Z-score maps obtained by the proposed method are shown in the left and middle columns, respectively. The images in the right column show the regions with a Z-score greater than 3. **a** Lung mass. **b** Left hilar lymph node. **c** Right breast mass

Figure 3 shows examples of Z-score maps obtained by our anomaly detection method. The proposed method can detect various lesions such as a lung mass, a hilar lymph node, and a breast mass in the same model. Note that the proposed method correctly enhances only the abnormal uptake foci and suppresses physiologic activity in the cardiovascular and abdominal regions.

**Fig. 4:**
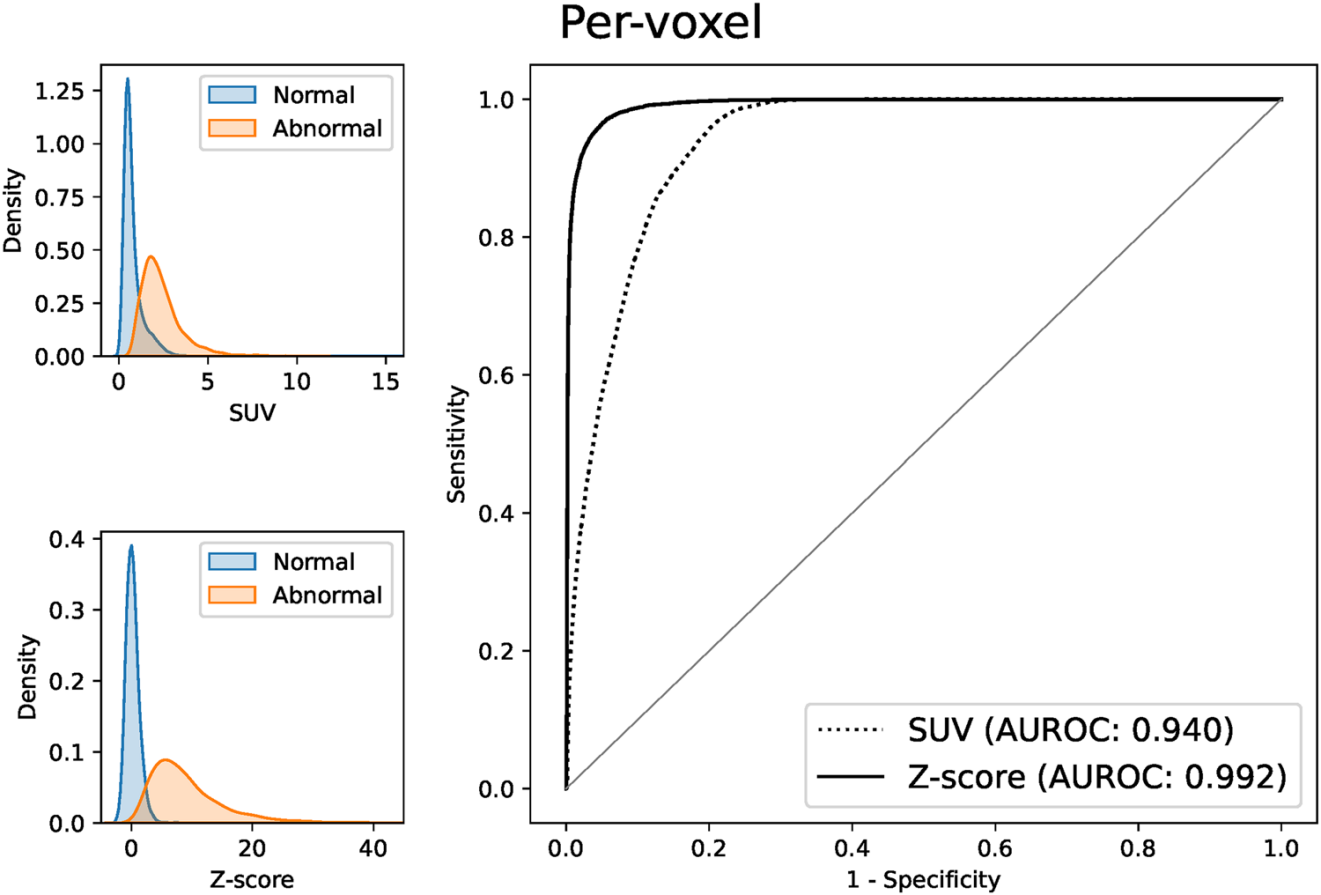
Results of per-voxel ROC analysis for our Z-score vs SUV. Left: density plots of our Z-score and SUV in normal and abnormal voxels. Right: ROC curves of our Z-score and SUV (AUROC 0.992 vs 0.940)

**Fig. 5:**
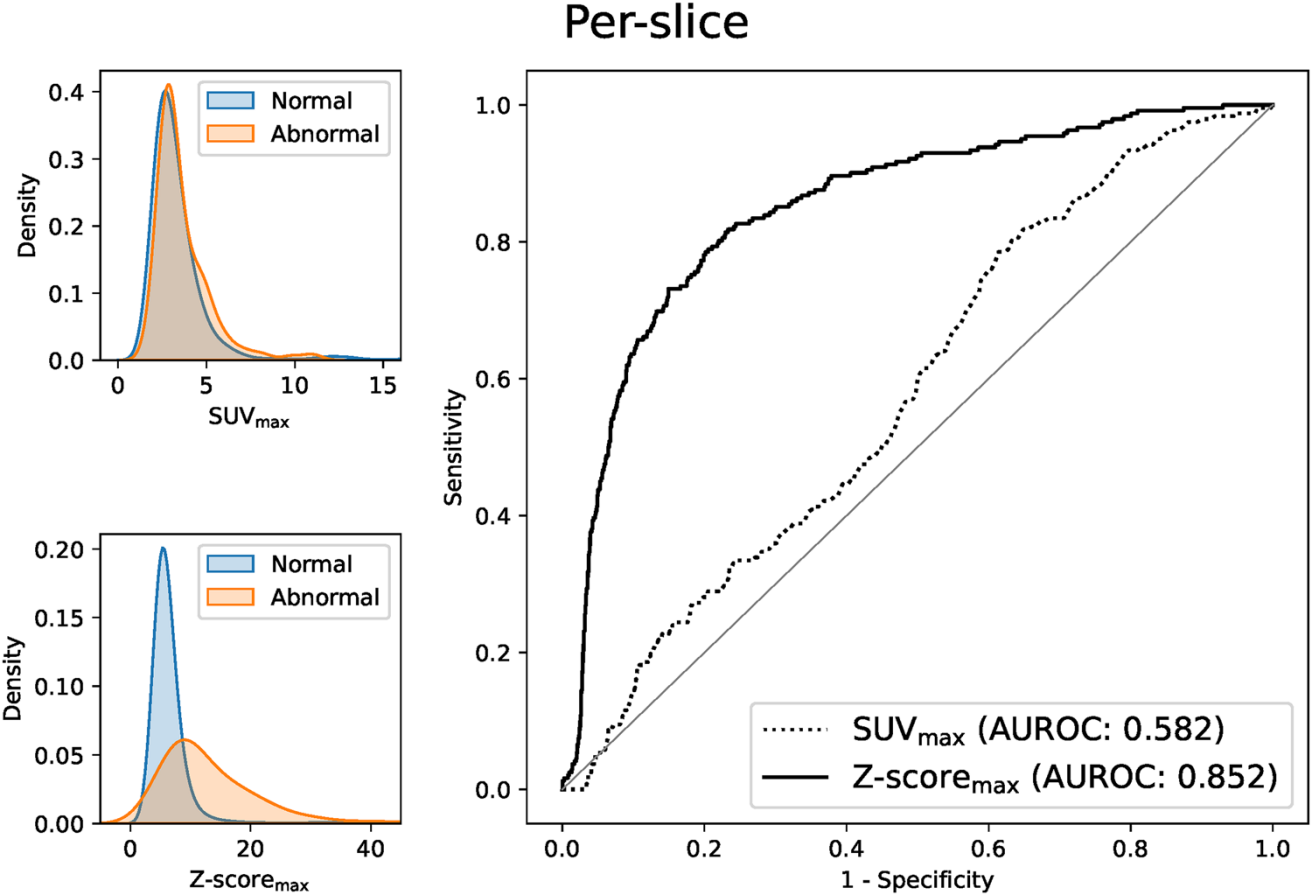
Results of per-slice ROC analysis for our Z-score vs SUV. Left: density plots of our Z-score_max_ and SUV_max_. Right: ROC curves of our Z-score_max_ and SUV_max_ (AUROC 0.852 vs 0.582)

Figure 4 shows the results of the per-voxel ROC analysis. Z score shows a better AUROC (area under the ROC curve) than SUV in discriminating between the normal and abnormal voxels (Z score: 0.992 vs SUV: 0.940). As shown on the left side of Fig. 4, SUVs in the normal voxels have a relatively long-tailed distribution to the right due to their variation among tissues, which causes some overlap of SUVs between the normal and abnormal voxels. On the other hand, Z scores in the normal voxels are more concentrated around zero and have less overlap between the normal and abnormal voxels. Results of the per-slice ROC analysis shown in Fig. 5 show this superiority of Z score clearer. Slice-level SUV_max_ shows almost the same distribution between the normal and abnormal slices and can hardly distinguish them (AUROC 0.582), whereas Z-score_max_ shows better discriminative performance (AUROC 0.852).

**Fig. 6:**
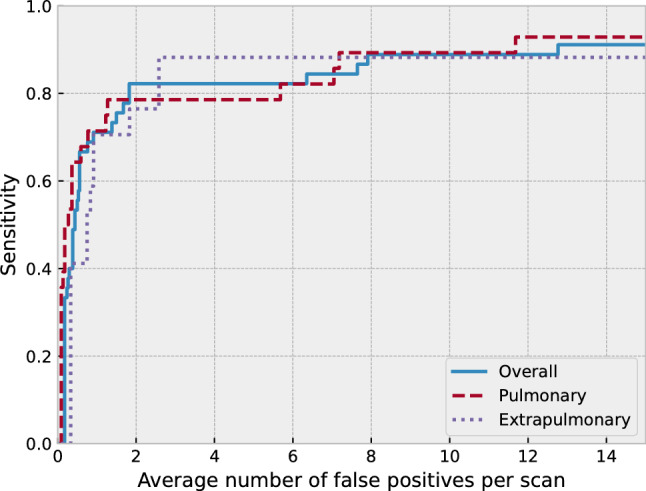
FROC curves of the proposed method. Our model detected 41 of 45 (91.1%) of the abnormal FDG uptake foci with 12.8 FPs/scan, which include 26 of 28 pulmonary and 15 of 17 extrapulmonary abnormalities. The sensitivity at 3.0 FPs/scan was 82.2% (37/45)

Figure 6 shows the FROC curves of the proposed method. Our model detected 41 of 45 (91.1%) of the abnormal FDG uptake foci with 12.8 false positives per scan (FPs/scan), which includes 26 of 28 (92.9%) pulmonary and 15 of 17 (88.2%) extrapulmonary abnormalities. The sensitivity at 3.0 FPs/scan was 82.2% (37/45). The four foci that were not detected were as follows: one lung mass, one pneumonia lesion, one clavicle fracture, and one breast mass. The lung mass could not be detected due to weak FDG uptake (SUV_max_ of 1.3), and the remaining three had high Z scores but were not well separated from the backgrounds.

**Fig. 7:**
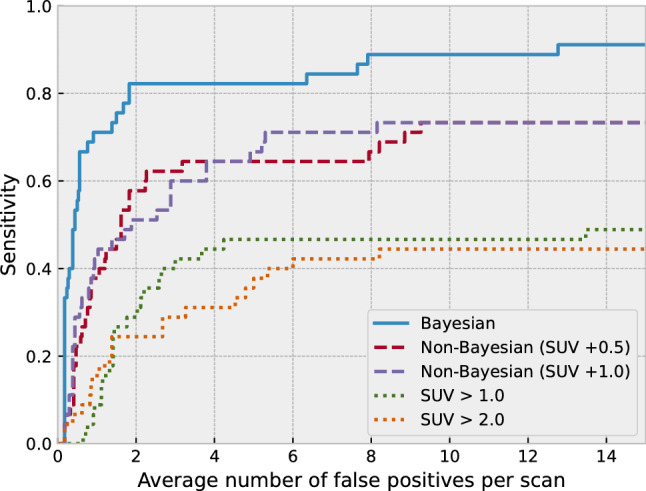
Performance comparison between the proposed method (Bayesian deep learning) and the baseline methods (non-Bayesian deep learning and simple SUV thresholding). The proposed method showed higher detection performance than the baseline methods

Figure 7 shows the performance comparison between the Bayesian and baseline methods. The proposed Bayesian method showed higher performance than the baseline methods.

We have shown that our anomaly detection method successfully detected abnormal FDG uptake foci in chest PET/CT images. We adopted an anomaly detection approach, which has two major advantages over PET/CT CAD studies with supervised learning [1–5]. The first is the ease of preparing the training dataset: the training requires only normal PET/CT images, and neither abnormal images nor lesion annotations are required. The second is the capability to detect lesions in various locations including both pulmonary and extrapulmonary regions. As mentioned in Introduction, the anomaly detection approach can detect any type of abnormality. Our results show that the proposed method has this capability.

Our method showed detection performance comparable to those developed in the previous studies of anomaly detection in PET or PET/CT [6–8] (Table 2). The main advantage of our method over them is whole-image anomaly detection in a simple way, which is derived from the use of deep learning. Previous studies based on machine learning techniques [7, 8] mainly utilized local features derived from CT values and SUVs. However, it is difficult to learn the variation in FDG uptake between organs only from such local features. To deal with this problem, in those studies, each detector targeted only a specific organ. In this case, abnormal uptake outside the target organs cannot be detected, which loses one of the advantages of anomaly detection, which can detect any type of abnormality. In another study [6], a nonrigid image registration of PET volumes to a standard human body atlas was performed. This anatomical standardization enables whole-body anomaly detection by voxel-wise comparison of the SUVs between images from the target patient and the healthy control group. However, this image registration requires a complicated, multi-step procedure. Such complex preprocessing may reduce the robustness of anomaly detection. Unlike these studies, in our method, both training and anomaly detection can be performed from the PET/CT images in raw form. This naturally provides whole-image anomaly detection, without requiring any complicated preprocessing. This capability to directly process high-dimensional data such as images is a great advantage of deep learning over conventional machine learning methods [16].

**Table 2:** Summary of previous anomaly detection studies using PET or PET/CT

	Modality	Organ(s)	Lesions	Performance
Kamesawa et al. [7]	PET/CT	Lung	Nodules, Pneumonia	Sensitivity of 81.9% with 5.0 FP lesion candidates per scan
Tanaka et al. [8]	PET/CT	Lung, neck, and mediastinum	(Not specified)	Sensitivities of 88.1% (right lung) and 87.5% (left lung) with 1,000 FP voxels per scan Sensitivity of 83.7% (neck and mediastinum) with 20,000 FP voxels per scan
Hara et al. [6]	PET	Whole body	Lesions from biopsy-proven malignant cases	417/432 (96.5%) lesions showed Z-score > 2.0 (FP not examined)

FP: false positive

Our results also show the usefulness of the Z-score approach using Bayesian deep learning. Our BNN learns the probability distribution of the SUVs instead of the SUVs themselves. This is a major difference from recent anomaly detection studies in other medical images [13, 18–22]. In these studies, image anomalies are typically detected by the difference, or absolute error, from the expected normal image. However, this absolute-error approach may not provide sufficient detection performance in PET images, since it cannot reflect the different widths of normal SUV ranges among organs. For example, an SUV of 1.0 higher than the normal average is almost certainly abnormal in the pulmonary region but may not necessarily mean abnormal in the myocardial region. Our results show that the Z-score approach based on Bayesian deep learning outperforms the absolute-error approach (Fig. 7 Bayesian vs non-Bayesian). The proposed method can only be applied to pairs of two anatomically matched images. Although the proposed method cannot be applied as is to general medical images, other pairs of functional and anatomical images such as PET/MRI and whole-body diffusion-weighted MRI meet this requirement and can be targets of the proposed method. We will investigate the application of our method to these modalities in our future work.

This study is a preliminary one and has the following limitations. First, the performance of our anomaly detection method was evaluated only for chest lesions in a relatively small number of images. To better demonstrate the usefulness of the proposed method, we are now preparing datasets containing various abnormalities found throughout the body. Second, this method cannot provide a qualitative diagnosis, such as whether the detected FDG uptake is from a malignant or a benign lesion. This is the limitation of the anomaly detection approach itself of learning the normal FDG distribution and detecting out-of-normal findings. In this sense, the proposed method will be suitable for initial screening, rather than for making a final diagnosis. Third, what our method detects is affected by the choice of the training dataset. For example, a bias of the training dataset towards older people will cause false-positive detections for the physiological findings specific in younger people, such as ovarian and endometrial uptake in premenopausal women. This problem may be addressed by the careful selection of training datasets depending on the target patients or by improving our method so that it can take clinical information such as age and gender into account. Finally, further performance improvements may be necessary before our proposed method can be used in clinical practice. The proposed method showed sufficient sensitivity in the lesion localization task, but it output up to approximately ten false-positive candidates per scan. A large number of false positives can lead users to neglect CAD outputs and impair the benefits of CAD, even with CAD’s high sensitivity [23]. Therefore, it is important to reduce the number of false positives while maintaining sensitivity. For example, employing a three-dimensional neural network or investigating more sophisticated postprocessing algorithms than simple Z-score thresholding may improve the detection performance.

In conclusion, our method based on a Bayesian deep learning technique successfully detected both pulmonary and extrapulmonary abnormalities in chest PET/CT images by training only with normal PET/CT images. In our future work, we plan to extend our target to whole-body PET/CT and also other modalities such as PET/MRI and whole-body diffusion weighted MRI.

## Figures and Tables

**Fig. 8:**
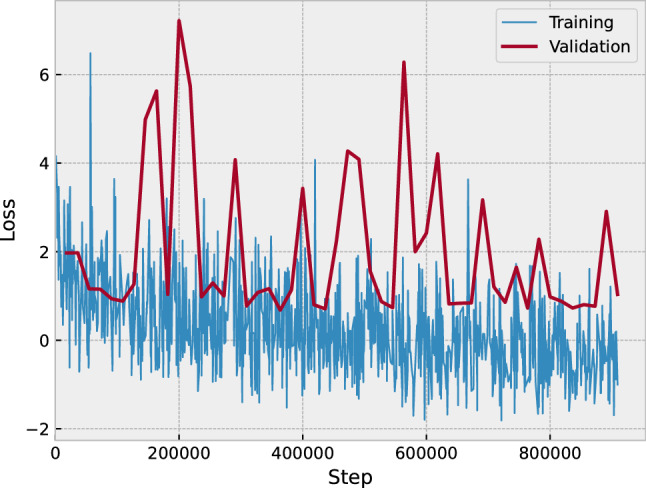
Training and validation losses of our BNN

**Fig. 9:**
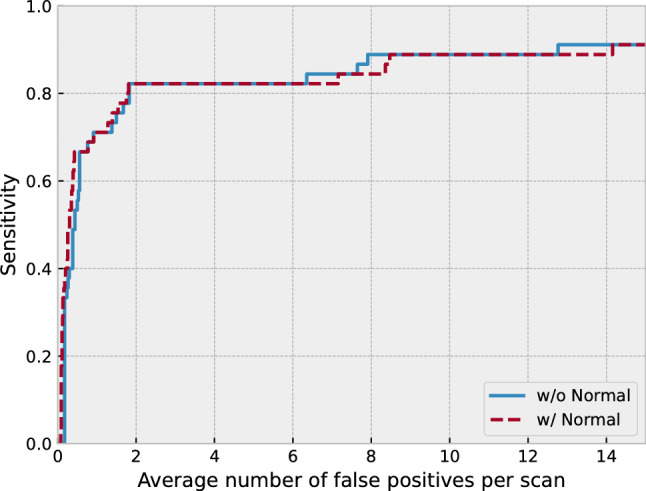
FROC analysis without (w/o) and with (w/) normal scans, which both showed almost the same performances. The curve “w/o” is the same as “Overall” in Fig. 6

## Appendix

### Training

Our BNN has a U-net [17] architecture as shown in Table

**Table 3:** Architecture of our U-net

Layer	#Channels	Output Size	BatchNorm	Dropout
(Input)	(1)	(256 × 256)	–	–
Conv1	64	128 × 128	No	No
Conv2	128	64 × 64	Yes	No
Conv3	256	32 × 32	Yes	No
Conv4	512	16 × 16	Yes	No
Conv5	512	8 × 8	Yes	No
Conv6	512	4 × 4	Yes	No
Conv7	512	2 × 2	Yes	No
Conv8	512	1 × 1	Yes	No
Deconv1	512	2 × 2	Yes	Yes
Deconv2	512	4 × 4	Yes	Yes
Deconv3	512	8 × 8	Yes	Yes
Deconv4	512	16 × 16	Yes	No
Deconv5	256	32 × 32	Yes	No
Deconv6	128	64 × 64	Yes	No
Deconv7	64	128 × 128	Yes	No
Deconv8	2	256 × 256	Yes	No

“Conv” and “Deconv” denote a convolutional and a transposed convolutional layer, respectively, both with a kernel size of 4 × 4 and a stride of 2. Every layer other than the last is followed by a Leaky Rectified Linear Unit (ReLU) activation function with a 0.2 slope

3. Its input is a two-dimensional CT slice and its output is a two-channel image, which consists of a predicted PET slice and a pixel-wise variance image. As in ref. [14], this BNN is trained using normal PET/CT images by maximum likelihood estimation. Assuming that each pixel value (SUV) of the PET slice follows a Gaussian distribution, the network can be trained by minimizing the following objective function $\mathcal{L}\left(\theta \right)$, which is derived from the negative log-likelihood of Gaussian distribution:

2 $$\rm{\mathcal{L}}\left( \theta \right) = \frac{1}{D}\sum_i {\left( {\frac{{\left( {y_i - \hat{y}_i } \right)^2 }}{{2\hat{\sigma }_i ^2 }} + \frac{1}{2}{\text{log}}\hat{\sigma }_i ^2 } \right),}$$

where $\theta$ is the network weights, $D$ is the number of output pixels, ${y}_{i}$ is the $i$-th pixel of the ground-truth PET image, ${\widehat{y}}_{i}$ is the $i$-th pixel of the predicted PET image, and ${{\widehat{\sigma }}_{i}}^{2}$ is the $i$-th pixel of the predicted variance image.

In the actual experiments, we employed a Laplace distribution instead of Gaussian, which is reported to provide better performance in regression tasks in vision [14]. We also found that network training progresses well by adding the L1 loss $\sum_{i}\left|{y}_{i}-{\widehat{y}}_{i}\right|$ to the objective function in addition to the above log-likelihood. The final objective function to minimize is:

3 $$\mathcal{L}\left(\theta \right)=\frac{1}{D}\sum_{i}\left(\frac{\sqrt{2}\left|{y}_{i}-{\widehat{y}}_{i}\right|}{{\widehat{\sigma }}_{i}}+\mathrm{log}{\widehat{\sigma }}_{i}\right)+\frac{\lambda }{D}\left(\sum_{i}\left|{y}_{i}-{\widehat{y}}_{i}\right|\right),$$

where λ is a hyperparameter that controls the contribution of L1 loss. We empirically set λ to 100 in the experiments.

The training was conducted for 50 epochs using the Adam optimizer [27] with a learning rate of 0.0002 and the momentum parameters β_1_ = 0.5 and β_2_ = 0.999. We used 1,800 out of the 1,878 training scans for the actual training of the BNN, and the remaining 78 for validation. We calculated the validation loss for the validation set at the end of each training epoch, and the parameters (network weights) with the minimum validation loss were used for the final evaluation. Figure 8 shows the learning curve of our BNN.

The non-Bayesian neural network for the baseline experiment had exactly the same U-net architecture as the Bayesian one, except that the number of output channel(s) was 1 instead of 2. This was trained to simply predict the PET slice from the corresponding CT slice using the L1 loss function.

### Inference

The inference is performed using a technique called Monte Carlo Dropout [14, 24]. Dropout [25] is a technique used to prevent neural networks from overfitting, which randomly sets the model weights to 0 during training. Here, dropout is also used during inference (test time) and the inference is performed $T$ times. From the $T$ sets of U-net outputs ${\left\{\widehat{{y}_{t}},{\widehat{{\sigma }_{t}}}^{2}\right\}}_{t=1}^{T}$, the mean and variance of normal SUVs can be estimated as

4 $$E({y}_{i})\approx \frac{1}{T} \sum_{t} {\widehat{y}}_{ti}$$

5 $$Var\left({y}_{i}\right)\approx \frac{1}{T} \sum_{t}{{\widehat{\sigma }}_{ti}}^{2}+\frac{1}{T} \sum _{t}{{\widehat{y}}_{ti}}^{2}-{E\left({y}_{i}\right)}^{2},$$

where $E({y}_{i})$ and $Var\left({y}_{i}\right)$ are the mean and variance of SUVs at the $i$-th pixel, and $\widehat{{y}_{ti}}$ and ${\widehat{{\sigma }_{ti}}}^{2}$ are the $i$-th pixel values of the $t$-th U-net outputs $\widehat{{{\varvec{y}}}_{{\varvec{t}}}}$ and ${\widehat{{{\varvec{\sigma}}}_{{\varvec{t}}}}}^{2}$ respectively.

### Lesion candidate extraction for FROC analysis

The lesion candidates in FROC analysis were extracted from Z-score maps by binary thresholding and connected-component analysis as follows,

1. Applying a median filter with a kernel size of 3
2. Masking the out-of-body area, which is automatically extracted from the CT image by the method of Nomura et al. [26]
3. Binary thresholding with Z > 3
4. Extracting connected components as lesion candidates

#### FROC analysis including normal scans

In the main text, FROC analysis was performed only for the abnormal scans. Here, to show that the tendency for false positives to be generated does not largely change for normal scans, we collected 61 additional normal scans (47 from males and 14 from females; mean age, 57.0 years; age range, 41–78 years) and added them to those for the evaluation for FROC analysis. These additional scans were collected similarly to those in the dataset described in the main text, from adults who visited our hospital between November 1st and 7th, 2015. Figure ^9^ shows that the addition of normal scans does not change the results significantly.
